# Immunology and Cell Biology of Parasitic Diseases 2014

**DOI:** 10.1155/2015/101025

**Published:** 2015-05-20

**Authors:** Luis I. Terrazas, Abhay R. Satoskar, Miriam Rodriguez-Sosa, Abraham Landa-Piedra

**Affiliations:** ^1^Unidad de Biomedicina, Facultad de Estudios Superiores-Iztacala, Universidad Nacional Autónoma de México, Avenida de Los Barrios No. 1, Los Reyes Iztacala, 54090 Tlalnepantla, MEX, Mexico; ^2^Departments of Pathology and Microbiology, Starling Loving Hall M418, The Ohio State University, Columbus, OH 43210, USA; ^3^Departamento de Microbiología y Parasitología, Facultad de Medicina, Universidad Nacional Autónoma de México, Circuito Escolar S/N, Ciudad Universitaria, 04510 México, DF, Mexico

Lack of clean water sources, starvation, insufficient hygiene, and poverty are some of the greatest barriers to health for the world's growing population. All these features are closely associated with parasitic infections. Approximately one third of the world's population has been infected with parasites at some point in their lives and these infections are often life-threatening. Since most parasitic diseases progress with few to no symptoms, patients do not obtain accurate diagnosis and treatment in a timely manner. Further, there are currently no accessible and effective antiparasitic vaccines despite enormous efforts and monetary investment into the development of vaccines, drugs, and treatments to combat these infections. Today parasitologists are looking for new alternatives for treatment such as immunotherapies, gene manipulation, or transfection in order to improve their fight against these “elusive” organisms. It is also clear that more studies on various parasites are necessary, even those which have low incidence, so that we are well-prepared during threats of reemerging parasitic infections.

In this special issue, we bring together several reviews as well as original reports that are intended to provide a summary of some of the current knowledge regarding the “immunology and cell biology of parasitic diseases.” These papers include basic, clinical, and epidemiologic studies that we believe are interesting and very important in our field. The first section of this special issue is focused on helminthic diseases ranging from vaccine development to helminth therapy and includes some research on the basic mechanisms of susceptibility, modulation, and protection against these parasites.

The work by D. M. Lopes et al., “Dendritic Cell Profile Induced by* Schistosoma mansoni* Antigen in Cutaneous Leishmaniasis Patients,” describes how helminth-derived molecules from* S. mansoni* can modulate dendritic cell activities during* Leishmania* infection. Additionally, V. H. Salazar-Castañon et al. have written an interesting review on how different helminth infections can improve or worsen the development of malaria in “Helminth Parasites Alter Protection against* Plasmodium* Infection.” Data from their epidemiological and experimental studies indicates that helminth infections are a double-edged sword, in the context of malaria.

The next series of papers are focused on the biology of helminths. L. Jiménez et al. characterize the thioredoxin-1 gene and gene product from* T. solium* in their original paper “Characterization of a Thioredoxin-1 Gene from* Taenia solium* and its Encoding Product.” Thioredoxin-1 is an essential component of the thioredoxin system and it performs functions such as antioxidative, protein-reducing, and signal-transducing ones for development, proliferation, migration, apoptosis, inflammation, and metabolism. It is secreted by* T. solium* and is able to modify the immune response by driving a Th2 biased response and allowing for the establishment of this parasite. Further, E.-V. Marcela et al. studied how the metacestodes of* T. crassiceps* “communicate” when they grow in vitro in a crowded manner. Their paper is entitled “Crosstalk among* Taenia crassiceps* (ORF Strain) Cyst Regulates Their Rates of Budding by Ways of Soluble and Contact Signals Exchanged between Them.”

Next, we put together a series of papers related to the development and regulation of immune responses to different helminths. K. E. Nava-Castro et al. demonstrated how early exposure to estrogens can imprint the immune response and have a positive or negative outcome during adulthood. Their results can be found in the paper “Diethylstilbestrol Exposure in Neonatal Mice Induces Changes in the Adulthood in the Immune Response to* Taenia crassiceps* without Modifications of Parasite Loads.” Using the same model, M. Khumbatta et al. show the important role that somatostatin has on the immune response and susceptibility to experimental cysticercosis in their paper “Somatostatin Negatively Regulates Parasite Burden and Granulomatous Responses in Cysticercosis.” A couple more papers focused on the immune response elicited by different helminth parasites. A. Prasad et al. report the first advances on immune response to the flat worm* Paramphistomum epiclitum* in their contribution “Evaluation of Antibody Response to Various Developmental Stage Specific Somatic Antigens of* Paramphistomum epiclitum* in Goats.” Y. Gu et al. report a promising experimental vaccine against trichinellosis in their paper “Protective Effect of a Prime-Boost Strategy with the Ts87 Vaccine against* Trichinella spiralis* Infection in Mice.” We have another paper on immunoregulation by helminths in the contribution of Y. Ledesma-Soto et al., “Extraintestinal Helminth Infection Limits Pathology and Proinflammatory Cytokine Expression during DSS-Induced Ulcerative Colitis: A Role for Alternatively Activated Macrophages and Prostaglandins.” This paper suggests that helminth infections can modulate intense inflammatory processes such as colitis through different mechanisms and ameliorate signs of illness in mice.

Finally in this helminth section, B. Moguel et al. show in their review “Transfection of Platyhelminthes” how knowledge of the genome of helminths and genetic manipulation can be useful for designing new and more effective antihelminthic drugs. They also explain the molecular crosstalk that occurs between the host and parasite which has been partly inaccessible to experimentation.

The second section of this special issue deals with protozoan infections which are widely spread around the world. We started this section with an old “friend” from developing countries, the amoeba, which causes amebiasis and remains as a public health problem in this part of the world. A. Aceves-Cano et al. present an original research “Morphological Findings in Trophozoites during Amoebic Abscess Development in Misoprostol-Treated BALB/c Mice,” where they show how trophozoites are altered with this kind of treatment. At human level A. K. Verma et al. contributed with the paper “The Trend in Distribution of Q223R Mutation of Leptin Receptor Gene in Amoebic Liver Abscess Patients from North India: A Prospective Study,” they show that heterozygous mutant (QQ versus QR, *P* = 0.049) and homozygous mutant (QQ versus RR, *P* = 0.004) were significantly associated with amoebic liver abscess when compared with homozygous wild type (QQ).

D. M. Meneses-Ruiz et al. developed a new experimental vaccine focused on the expression of a lectin in their paper “Protection against Amoebic Liver Abscess in Hamster by Intramuscular Immunization with an* Autographa californica* Baculovirus Driving the Expression of the Gal-Lectin LC3 Fragment,” where they found up to 75% protection using this novel vaccine compared to control animals. In the clinical field, we have a couple of papers related to amebiasis. The first paper is by L. R. Iyer et al. and is entitled “Differential Expression and Immunolocalization of Antioxidant Enzymes in* Entamoeba histolytica* isolates during Metronidazole Stress.” Their work reports the behavior of the antioxidant enzymes during metronidazole stress on the strain HM1: IMSS versus clinical isolates of* Entamoeba histolytica* from India. Metronidazole is indiscriminately used in India. Their results revealed that the Indian isolate could tolerate higher concentrations of the drug compared to standard strains. Thus, the authors found an alarming resistance to metronidazole in the* Entamoeba* isolates from India. We have some more novel work with amoeba presented by Y. Toledano-Magaña et al., who used the highly phagocytic activity of* E. histolytica* as a tool to study the effects of new nanomaterials in their report “Effect of Clinoptilolite and Sepiolite Nanoclays on Human and Parasitic Highly Phagocytic Cells.” They found that these nanomaterials were well tolerated by macrophages from human, mice, and the RAW 264.7 cell line as well as in* E. histolytica* trophozoite cultures. The final paper on amebiasis is an interesting comparative study between two species of amoebas:* Entamoeba histolytica* (pathogenic strain) and* E. dispar* (a noninvasive strain). Talamás-Lara et al. demonstrated a dramatic difference in the ability to phagocyte in these two species in their paper, “Erythrophagocytosis in* Entamoeba histolytica* and* Entamoeba dispar*: A Comparative Study.” They discovered that* E. histolytica* displayed a superior erythrophagocytosis activity which possibly contributes to its more pathogenic nature.

Other protozoan parasites included in this special issue are* Trypanosoma*,* Leishmania*, and* Plasmodium*.

A. Y. Cervantes-Landín et al. contributed their work “High Molecular Weight Proteins of* Trypanosoma cruzi* Reduce Cross-Reaction with* Leishmania* spp. in Serological Diagnosis Tests.” The aim of this study was to improve serological tests already standardized for Chagas disease diagnosis, by using a high molecular weight protein fraction from* T. cruzi* extracts. They developed an easier and cheaper assay which is much needed in poor countries that suffer from this debilitating disease. From the same group, A. Vizcaíno-Castillo et al. describe the processes of acute inflammation derived after experimental* T. cruzi* infection in their original paper “Exacerbated Skeletal Muscle Inflammation and Calcification in the Acute Phase of Infection by Mexican* Trypanosoma cruzi* DTUI Strain.” In Leishmaniasis research, we have several interesting papers, such as “CK2 Secreted by* Leishmania braziliensis* Mediates Macrophage Association Invasion: A Comparative Study between Virulent and Avirulent Promastigotes” by A. M. B. Zylbersztejn et al., where the authors comparatively analyze the effect of the kinase enzyme CK2 of virulent and avirulent* L. braziliensis* strains on parasite growth and macrophage invasion. They show interesting data that demonstrates that CK2 has a critical influence as a mechanism of invasion used by* L. braziliensis*. On the clinical side, N. Verma et al. show important cases where immunological changes were observed according to the treatment carried out by the patients, in their work “Clinicopathological and Immunological Changes in Indian Post Kala-Azar Dermal Leishmaniasis (PKDL) Cases in relation to Treatment: A Retrospective Study.” In another review by S. A. G. Da-Silva et al. entitled “The Dialogue of the Host-Parasite Relationship:* Leishmania* spp. and* Trypanosoma cruzi* Infection,” they have analyzed the host-parasite interaction in both* Leishmania *spp. and* Trypanosoma cruzi* infections. This review is comprehensive and is interesting since it indicates that there are some differences in host invasion strategies between these two major protozoan parasites. Three schematic figures summarizing the main escape mechanisms of* Trypanosoma* and* Leishmania* and modulation of host cells are included.

Another interesting original study related to malaria is presented by N. A. Mosqueda-Romo et al. in “Gonadal Steroids Negatively Modulate Oxidative Stress in CBA/Ca Female Mice Infected with* P. berghei* ANKA,” where the authors highlight the important effect of sexual hormones on the development of* Plasmodium* infection.

Finally, a review of the basic cell biology of protozoan parasites and their virulence is discussed by C. Muñoz et al., in their latest paper, “Role of the Ubiquitin-Proteasome Systems in the Biology and Virulence of Protozoan Parasites.”

We believe that this compilation of original research as well as the latest reviews written by authors from all around the world is a small sample about interesting yet complicated field of immunoparasitology. This research shows us that it is necessary to develop a deeper knowledge about the different mechanisms that parasites employ to invade the host and to avoid antiparasitic immune responses. We also require a better understanding of how they develop resistance to constant exposure to old drugs.

*Luis I. Terrazas* *Abhay R. Satoskar* *Miriam Rodriguez-Sosa* *Abraham Landa-Piedra*

